# Alternative splicing regulates PACC1 function and promotes acidosis-induced cytotoxicity

**DOI:** 10.3389/fcell.2025.1754079

**Published:** 2026-01-30

**Authors:** Serena Tamburro, Giulia Gorrieri, Niccolò Callegari, Floriana Guida, Francesca Antonini, Ilaria Musante, Simona Baldassari, Federico Zara, Paolo Scudieri

**Affiliations:** 1 Department of Neurosciences, Rehabilitation, Ophthalmology, Genetics, Maternal and Child Health (DiNOGMI), University of Genoa, Genoa, Italy; 2 Medical Genetics Unit, IRCCS Istituto Giannina Gaslini, Genoa, Italy; 3 Core facilities for Omics Science, IRCCS Istituto Giannina Gaslini, Genoa, Italy

**Keywords:** alternative splicing, anion channel, cell death, isoform-specific function, RNAscope, spatial biology

## Abstract

**Introduction:**

*PACC1* (also named *TMEM206*) encodes a proton-activated chloride channel implicated in acid-induced cell death, but its tissue distribution, cellular expression, and isoform-specific roles are incompletely understood.

**Methods:**

We mapped PACC1 expression and splicing across normal human tissues, with emphasis on the central nervous system, using RNAscope *in situ* hybridization, quantitative cell-type-specific co-detection, RT-PCR, and isoform-specific *in situ* probes. Functional properties of PACC1 splice variants were assessed by reconstituting each isoform in PACC1-deficient cells.

**Results:**

PACC1 was broadly expressed across tissues, with especially high and uniform expression in the brain. Quantitative analyses revealed PACC1 localization in both neurons and astrocytes, with higher abundance in astrocytes. Two major splice variants, PACC1-V1 and PACC1-V2, were investigated, distinguished by exon 2 inclusion and exhibiting distinct tissue and developmental expression patterns. Functional assays indicated isoform-specific differences: PACC1-V2 predominantly localized to endosomes and prevented endosomal hyperacidification, whereas PACC1-V1 accumulated at the plasma membrane and enhanced acid-induced cell death.

**Conclusion:**

Alternative splicing governs PACC1 channel trafficking and function. Isoform-specific behavior suggests distinct roles for PACC1 variants in cell development and responses to acid stress, particularly within the nervous system.

## 1 Introduction

The tissue-, cellular-, and subcellular-specific localization of gene products is essential for understanding gene function in both physiological and pathological contexts. This principle underpins the emerging field of spatial biology, which integrates molecular profiling with spatial information to map biomolecules within their native tissue architecture (Seydel, 2025). Techniques like *in situ* hybridization (ISH), multiplexed immunofluorescence, and spatial transcriptomics or proteomics enable precise detection of RNA transcripts and proteins while preserving tissue and cellular morphology (Jamalzadeh et al., 2022; Kalhor et al., 2023; Liu et al., 2024). These approaches provide critical insights into spatial gene expression patterns, facilitating a deeper understanding of cellular differentiation, tissue organization, and the molecular mechanisms that govern health and disease.

*PACC1*, also referred to as *TMEM206*, encodes a proton-activated chloride channel (PAC) that is broadly expressed in human tissues. Since its discovery in 2019 (Ullrich et al., 2019; Yang et al., 2019), subsequent studies have begun to elucidate its molecular architecture and physiological roles. Structural models and cryo-EM data revealed that PAC assembles as a trimer, with each 350 aminoacid-long subunit containing two membrane spanning helices (TM1 and TM2), a large β-rich loop, and short N- and C-terminal tails (Ruan et al., 2020; Deng et al., 2021). Key structural elements include the highly conserved transmembrane segments that form the channel pore, the anion selectivity filter determined by the positively charged lysine-319 on TM2, and the proton sensing domain composed of titratable residues (histidines, glutamates, and aspartates) within the β-rich loops (Ruan et al., 2020; Deng et al., 2021; Osei-Owusu et al., 2022; Wang et al., 2022; Xu et al., 2023).

Functional studies conducted in various cell models suggested that PAC participates in a wide range of cellular processes, including acid sensing, ion transport across the plasma membrane and intracellular organelles, regulation of endosomal acidification, receptor-mediated endocytosis, macropinosome volume regulation, and acid-induced cell death (Ullrich et al., 2019; Yang et al., 2019; Osei-Owusu et al., 2021; Zeziulia et al., 2022). Upon activation by acidic pH, PAC modulates these cellular functions in a manner that is tightly linked to its subcellular distribution (Capurro et al., 2015; Osei-Owusu et al., 2021). Early studies focused on plasma membrane-localized PAC and its contribution to acidotoxicity through cell death pathways (Capurro et al., 2015; Ullrich et al., 2019; Yang et al., 2019; Osei-Owusu et al., 2020). Notably, PAC silencing nearly abolishes proton-activated chloride currents in both human and rodent cell lines and confers protection from extracellular acidosis-induced cell death, a pathological feature observed in conditions such as ischemia, cancer, infection, and inflammation (Ullrich et al., 2019; Yang et al., 2019; Osei-Owusu et al., 2020). More recent studies have delineated a critical role for PAC within the endocytic pathway, implicating it in intracellular trafficking and vesicular pH regulation (Osei-Owusu et al., 2021; Zeziulia et al., 2022). In addition to being present at the cell surface, PAC channel traffics to early and recycling endosomes via a canonical YxxL motif in its N-terminal tail (YQEL sequence at residues 10-13) (Osei-Owusu et al., 2021). Within endosomes, PAC functions as a chloride leak channel, acting as a brake to prevent excessive luminal acidification. Accordingly, PAC deletion leads to hyper-acidification of endosomes, while PAC overexpression causes endosomal alkalinization (Osei-Owusu et al., 2021). Endosomal PAC negatively regulates transferrin-mediated endocytosis and positively regulates AMPA receptor endocytosis underlying synaptic plasticity, especially during long-term depression in neurons (Osei-Owusu et al., 2021; Chen et al., 2025). PAC is also internalized during macropinocytosis, where its pH-dependent activity governs the volume, sorting and trafficking of macropinosomes (Zeziulia et al., 2022). In macrophages, PAC deletion impairs macropinosomes resolution (shrinkage), disrupts receptor recycling, and ultimately diminishes C5a-induced cell migration (Zeziulia et al., 2022).

Together, these findings underscore PAC’s multifaceted roles in cell physiology, which are tightly governed by its cellular and subcellular localization. However, despite its broad expression and functional relevance, a comprehensive spatial profile of *PACC1* in human tissues is still lacking. Furthermore, the molecular mechanisms regulating *PACC1* expression across different tissues and cell types remain completely unknown.

In this study, using multiplex RNAscope on human tissue microarrays, we identified distinct *PACC1* expression patterns, with the brain exhibiting the highest and most homogeneous expression, notably in both neuronal and glial cells. We characterized an alternatively spliced *PACC1* isoform (PACC1-V1), featuring exon 2 inclusion, which is selectively expressed in few fetal (brain and smooth muscle) and adult (brain and heart) tissues. PACC1 splice protein isoforms displayed differential subcellular localization and functional roles, including effects on endosomal pH and plasma membrane anion transport. Cells expressing PACC1-V1 were more susceptible to acid-induced cell death. These findings highlight the critical role of spatial and isoform-specific regulation of *PACC1* in cellular physiology and suggest relevant implications in acid-related pathologies.

## 2 Materials and methods

### 2.1 RNAscope *in situ* hybridization (ISH)

Paraffin-embedded multi-organ (DBA, BN1021) and normal brain (DBA, BNC17011c) tissue microarray were processed and stained using the RNAscope™ Multiplex Fluorescent Reagent Kit v2 (Bio-Techne, 323100) according to the manufacturer’s protocol with protease-based sample pretreatment. Slides were hybridized with the probes and fluorophores listed in Table 1, and then mounted with Fluoroshield with DAPI (Merk, F6057) and glass coverslips. To confirm probe specificity, standard RNAscope controls were performed, including positive control probes targeting housekeeping genes with low (*POLR2A*) and high (*UBC*) expression, and RNase pretreatment to verify signal dependence on RNA integrity and probes specificity. Representative control data are provided in Supplementary Figure S1.

**TABLE 1:** RNAscope probes and fluorophores.

Reagent	Dilution	Company Cat #
RNAscope™ probe - Hs-TMEM206	1x	Bio-Techne, 456451
RNAscope™ probe - Hs-S100B-C2	1x	Bio-Techne, 430891-C2
RNAscope™ probe - Hs-RBFOX3-C3	1x	Bio-Techne, 415591-C3
TSA Vivid™ fluorophore 520	1:3,000	Bio-Techne, 323271
TSA Vivid™ fluorophore 570	1:3,000	Bio-Techne, 323272
TSA Vivid™ fluorophore 650	1:5,000	Bio-Techne, 323273

cat # stand for catalog number.

### 2.2 mRNA and protein co-detection

Paraffin-embedded normal brain tissue microarrays (DBA, BNC17011c) were processed and stained using the RNAscope™ Multiplex Fluorescent Reagent Kit v2 (Bio-Techne, 323100) according to the manufacturer protocol with the protease-free sample pretreatment and RNAscope Multiplex Fluorescent v2 Assay combined with immunofluorescence. We used the RNAscope™ Probe-Hs-TMEM206 (Bio-Techne, 456451) and the primary and secondary antibodies listed in Table 2 to detect MAP2 and GFAP proteins.

**TABLE 2:** Primary and secondary antibodies.

Antibody	Dilution	Company Cat #
Mouse IgG2a anti-GFAP	1:100	Synaptic systems, 173011
Guinea pig anti-MAP2	1:100	Synaptic systems, 188004
Mouse anti-HA	1:1,000	BioLegend, 901514
Rabbit anti-HA	1:1,000	Abcam, ab9110
Rabbit anti-calnexin	1:1,000	Abcam, 22595
Rabbit anti-TGN46	1:1,000	Abcam, 50595
Mouse anti Na^+^/K^+^ ATPase	1:200	Merck, 05-369
Mouse anti-EEA1	1:250	BD biosciences, 610457
Rabbit anti-RAB7	1:100	Cell signaling, 9367S
Mouse anti-transferrin	1:500	Thermo Fisher Scientific, 13-6800
Rabbit anti-LAMP1	1:1,000	Abcam, 24170
Mouse anti-GAPDH antibody	1:1,000	Merck, MAB374
Alexa Fluor™ 647 goat anti-mouse IgG2a	1:200	Thermo Fisher Scientific, A21241
Alexa Fluor™ 488 goat anti-Guinea pig	1:200	Thermo Fisher Scientific, A11073
Alexa Fluor Pus™ 555 goat anti-mouse IgG	1:500	Thermo Fisher Scientific, A32727
Alexa Fluor™ 555 goat anti-rabbit	1:500	Thermo Fisher Scientific, A21428
HRP anti-mouse secondary antibody	1:10,000	Agilent DAKO, P0447

cat # stand for catalog number.

### 2.3 RNAscope images acquisition and analysis

Image acquisition was performed using a TCS SP8 laser scanning confocal microscope (Leica Microsystems). Image analysis was carried out using the General Analysis module in NIS-Elements AR software. Following background subtraction, an intensity threshold was applied to the nuclear channel to detect and count nuclei. To segment and define individual cells, the binary processing tools “grow bright regions to intensity,” “contour,” and “remove objects touching borders” were applied to the nuclei binary mask. For RNAscope signal detection, background subtraction and thresholding were first performed on the corresponding channel. Real-time tuning of thresholding window identified the minimum RNAscope dot size as 6 μm^2^. For quantification of target mRNA molecule per cell, the total RNAscope signal area within each defined cell was measured and divided by the area of a single dot (6 μm^2^), yielding the number of estimated dots (eDots) per cell. This approach enabled accurate estimation of mRNA transcript numbers even in the presence of signal clusters, which are typically observed in cells with high target mRNA abundance.

### 2.4 RT-PCR analysis

Commercially available cDNA panels derived from various human tissues pooled from multiple donors (636742, Human MTC panel I; 636743, Human MTC panel II; 636747, Human Fetal MTC Panel; all from Takara Bio) were used according to manufacturer’s instructions to perform PCR with the AmpliTaq Gold™ 360 Master Mix (Thermo Fisher Scientific, 4398881). To detect the expression of the two *PACC1* isoforms, primers flanking exon 2 were used: forward primer 5′-GTA​GGT​CCA​GGT​GCA​GCG-3′ and reverse primer 5′-CAG​CTC​TGA​GTT​CTC​AAC​CA-3’. The housekeeping gene *G3PDH* was detected using the following primers: forward primer 5′- TGA​AGG​TCG​GAG​TCA​ACG​GAT​TTG​GT-3′ and reverse primer 5′- CAT​GTG​GGC​CAT​GAG​GTC​CAC​CAC-3’.

### 2.5 BASEscope *in situ* hybridization (ISH)

Paraffin-embedded normal brain tissue microarrays (DBA, BNC17011c) were processed and stained using the BaseScope™ Duplex Detection Reagent Kit (Bio-Techne, 32300) following the manufacturer’s instructions. To specifically detect exon 2-containing PACC1 transcripts, we used the BaseScope™ probe BA-Hs-PACC1-tv1-E2-3zz-st-C1 (Bio-Techne, 1210631-C1), which targets base pairs 166-286 of the NM_001198862.2 transcript. Slides were scanned using a Nikon Eclipse Ti automated microscope equipped with a × 20 objective and color camera.

### 2.6 HEK-293 cell culture and transfection

Parental and *PACC1*-knockout (KO) HEK293 cells were obtained from Ubigene (YKO-H052) and maintained in DMEM/Ham’s F-12 medium supplemented with 10% fetal bovine serum, 2 mM L-glutamine, 100 U/mL penicillin, and 100 μg/mL streptomycin. *PACC1* knockout was validated by PCR and Sanger sequencing using primers flanking the targeted region in exon 3 of the *PACC1* coding sequence (forward primer: 5′-TGA​CAA​TTA​AGT​GAC​AAG​CAG​CT-3’; reverse primer: 5′-GCT​CCT​CCT​CTC​CCA​CAG-3′).

For Western blot analysis, *PACC1*-KO HEK-293 cells were seeded at a density of 500,000 cells per well in 6-well plates. After 24 h, cells were transfected with plasmids encoding either PACC1-V1 or PACC1-V2 containing a C-terminal triple HA, using Lipofectamine™ 2000 Transfection Reagent (Thermo Fisher Scientific, 11668027) in Opti-MEM™ (Thermo Fisher Scientific, 51985026). Each transfection used 1 µg of plasmid DNA and 5 µL of Lipofectamine per well.

For immunofluorescence, *PACC1*-KO HEK-293 cells were seeded at 25,000 cells per well in 8-well chamber slides (Ibidi, 80841). Cells were transfected 24 h later with 0.1 µg of plasmid DNA and 0.5 µL of Lipofectamine per well. After 24 h, the transfection medium was replaced with fresh culture medium.

For endosomal pH measurements, *PACC1*-KO HEK-293 cells were seeded at 300,000 cells per well in 12-well plates pre-coated with Poly-D-Lysine (Gibco, A3890401) and transfected after 24 h with plasmids encoding PACC1-V1 or PACC1-V2 tagged with C-terminal mCherry. Transfection was performed using Lipofectamine™ 2000 in Opti-MEM™, with 0.5 µg of plasmid DNA and 2.5 µL of Lipofectamine per well. Medium was replaced 24 h post-transfection.

For the functional assay based on the halide-sensitive yellow fluorescent protein (HS-YFP) (Caputo et al., 2008; Scudieri et al., 2015, Scudieri et al., 2016), parental and *PACC1*-KO HEK-293 cells were seeded at a density of 125,000 cells/cm^2^ in black 96-well plates (Corning, 3,603) pre-coated with Poly-D-Lysine (Gibco, A3890401). After 24 h, cells were co-transfected with HS-YFP and either PACC1-V1 or PACC1-V2 constructs tagged with mCherry, using Lipofectamine™ 2000 in Opti-MEM™. For each well, we used 0.2 μg of total plasmid DNA and 0.5 μL of Lipofectamine. Each well received 0.2 µg of total plasmid DNA and 0.5 µL of Lipofectamine. Twenty-four hours post-transfection, the medium was replaced with fresh culture medium.

For the acid-induced cell death assay, HEK-293 cells were seeded and transfected following the same procedure as for the HS-YFP assay, except that PACC1 constructs tagged with triple-HA were used.

### 2.7 Western blot analysis

Transfected *PACC1*-KO cells were lysed 48 h after transfection in lysis buffer (NaCl 150mM; tris HCL pH7,4 50 mM, EDTA 1 mM and triton X 1%) supplemented with a protease inhibitor cocktail (complete EDTA-free protease inhibitors, Roche, 1187358001). Protein concentration was quantified using DC™ Protein Assay Kit II (Bio-Rad, 5000112). Equal amounts of total protein (15 µg per sample) were separated on 4%–15% SDS-PAGE pre-casting gel (Bio-Rad, 4561083) and transferred onto a nitrocellulose membrane (Bio-Rad, 1704158). PACC1 protein was detected using a monoclonal mouse anti-HA antibody (BioLegend, 901514) at 1:1,000 dilution, followed by an HRP (horseradish peroxidase)-conjugated anti-mouse secondary antibody Agilent DAKO, P0447) at 1:10,000 dilution. Membranes were subsequently stripped using Restore Western blot Stripping Buffer (Thermo Fisher Scientific, 21059) and reprobed with anti-GAPDH antibody (Merck, MAB374) as a loading control. All antibodies were dissolved in 5% (w/v) non-fat dried skimmed milk powder in TBS-T (Tris-buffered saline with Tween 20). Protein bands were visualized using the Clarity Western ECL substrate (Bio-Rad, RPN2106). Chemiluminescence was recorded directly using the Mini HD9 system (Uvitec Cambridge). Densitometric analysis was performed with Fiji (ImageJ). Uncropped full-length images of the Western blots presented in Figure 5 are provided in the Supplementary Material.

### 2.8 Immunofluorescence analysis

*PACC1*-KO HEK293 cells transfected with either PACC1-V1 or PACC1-V2 tagged with a C-terminal triple HA epitope were washed in PBS and fixed with 200 μL of 10% neutral buffered formalin (Bio-Optica, 05-01005Q) for 5 min at room temperature. Following three washes in PBS, cells were permeabilized with 0.3% Triton X-100 in PBS for 5 min, then blocked with 1% BSA in PBS for 2 h at room temperature. Cells were then incubated overnight at 4 °C with 200 µL of primary antibody solution prepared in PBS containing 1% BSA. The solution contained either a mouse or rabbit anti-HA antibody, in combination with antibodies targeting organelle-specific markers. Detailed antibody information and dilutions are provided in Table 2. After primary antibody incubation, cells were rinsed three times with PBS and incubated for 1 h in the dark with 200 µL of Alexa Fluor–conjugated secondary antibodies (Thermo Fisher Scientific), diluted 1:500 in PBS with 1% BSA. After three additional PBS washes, chambers were removed, and slides were mounted using Fluoroshield mounting medium with DAPI (Merck, F6057) to counterstain nuclei.

Image acquisition was performed using a TCS SP8 laser scanning confocal microscope (Leica Microsystems). For co-localization analysis, individual cells were cropped, and Pearson’s correlation coefficient (R) above threshold was calculated using the Coloc2 plugin in Fiji (ImageJ). Pearson’s R values were quantified for 15-25 individual cells per condition from three independent transfections. Additionally, co-localization was assessed by calculating the proportion of organelle-stained structures overlapping with PACC1 signal (detected via anti-HA) using the General Analysis module in NIS-Elements AR software.

### 2.9 Endosomal pH assay

Endosomal pH was measured using an assay based on the uptake of fluorophore-conjugated transferrin (Osei-Owusu et al., 2021). Parental and *PACC1*-KO transfected HEK-293 cells were incubated for 30 min at 37 °C in serum-free medium to remove residual transferrin, followed by incubation for 1 h at 37 °C with a mixture of 75 μg/mL FITC-conjugated transferrin (pH-sensitive, Thermo Fisher Scientific, T2871) and 25 μg/mL Alexa Fluor 633-conjugated transferrin (pH-insensitive, Thermo Fisher Scientific, T23362). To halt further transferrin uptake, cells were placed on ice and washed twice with cold PBS (pH 7.4) to remove excess transferrin. This was followed by a wash with cold PBS at pH 5 to remove surface-bound transferrin, and a final wash with cold PBS (pH 7.4). Cells were then detached using trypsin and analyzed by flow cytometry on a Beckman Coulter CytoFLEX SRT, acquiring data from 10,000 cells per condition and replicate. To calculate absolute endosomal pH values, a standard curve was generated using wild-type HEK-293 cells loaded with the same fluorescent probes. After probe uptake, these cells were resuspended in calibration buffers of known pH (7.4, 6.5, 5.5, and 4.5) and permeabilized with 10 µM valinomycin and 10 µM nigericin for 5 min at 37 °C. This permeabilization equilibrated intracellular pH with the extracellular buffer. Experimental sample fluorescence ratios were then interpolated against this standard curve to determine their corresponding endosomal pH values.

### 2.10 Halide-sensitive (HS) YFP assay

The HS-YFP quenching assay was performed 48 h post-transfection. HEK cells on 96-well plates were washed with PBS (Dulbecco’s PBS containing Calcium & Magnesium, Euroclone, ECB4053L), incubated with 60 µL of PBS for 20 min, and then transferred to a microplate reader (FluoStar Galaxy; BMG Labtech, Offenburg, Germany). The plate reader was equipped with high-quality excitation (HQ500/20X: 500 ± 10 nm) and emission (HQ535/30M: 535 ± 15 nm) filters for YFP (Chroma Technology). Each assay consisted of continuous fluorescence acquisition for 14 s, with 2 s recorded before and 12 s after injection of 165 μL of iodide-containing buffer (PBS-NaI, Table 3) adjusted to different pH values. Data were background-subtracted and normalized to initial fluorescence values. To determine the rate of YFP fluorescence quenching, indicative of anion influx, the final 10 s of data for each well were fitted with an exponential function to extract the initial slope (dF/dt). To validate results from the plate reader, single-well imaging experiments were also performed using a Nikon Eclipse Ti microscope. Cells were incubated with 60 µL of PBS, imaged first for mCherry fluorescence, and then subjected to time-lapse acquisition of HS-YFP fluorescence during manual injection of 165 µL of PBS-NaI at varying pH values. Quenching rates were determined in mCherry-positive cells and showed no significant differences compared to those obtained from bulk well measurements.

**TABLE 3:** Composition of PBS-NaI solution.

Reagent	mM	Company Cat #
NaI	137.00	Supelco, 1.06404.5000
KCl	2.70	Supelco, 1.04936.0500
Na_2_HPO_4_	8.10	Merck, S9638-500G
KH_2_PO_4_	1.50	Merck, P0662-500G
CaCl_2_	1.00	Merck, C5670-100G
MgCl_2_	0.50	Merck, M8266-100G

cat # stand for catalog number.

### 2.11 Acid-induced cell death assay

Twenty-four hours post-transfection, cells were treated for 2 h at 37 °C with a solution containing NaCl (145 mM), KCl (5 mM), MgCl_2_ (1 mM) and CaCl_2_. (2 mM), and glucose (10 mM) adjusted to different pH values (7.4, 5.2, 4.5, and 4.0). Following treatment, the solutions were replaced with pH 7.4 buffer containing Hoechst 33342 (1 μg/mL) and propidium iodide (PI) (5 μg/mL), and cells were incubated for 15 min at 37 °C. Cells were then washed three times with PBS, fixed with 10% neutral buffered formalin (Bio-Optica, 05-01005Q), and prepared for imaging.

Image acquisition was performed using an automated Nikon Eclipse Ti microscope, and image analysis was conducted with the General Analysis module in NIS-Elements AR software (Nikon) to detect and quantify PI-positive nuclei.

### 2.12 Quantification and statistical analysis

Data are shown as representative images and quantitative graphs reporting single data points and/or mean ± SEM obtained from different biological replicates. The precise number of biological replicates for each experiment are indicated in figure legends. To assess significant differences between groups of data, we used the Kolmogorov–Smirnov test to assess normal distribution and then used 2-tailed Student’s t-test to compare PACC1-V1 and PACC1-V2 or one-way ANOVA (followed by Tukey’s *post hoc* test), for more than 2 groups.

## 3 Results

### 3.1 Expression profiling of PACC1 in human tissues

**FIGURE 1:**
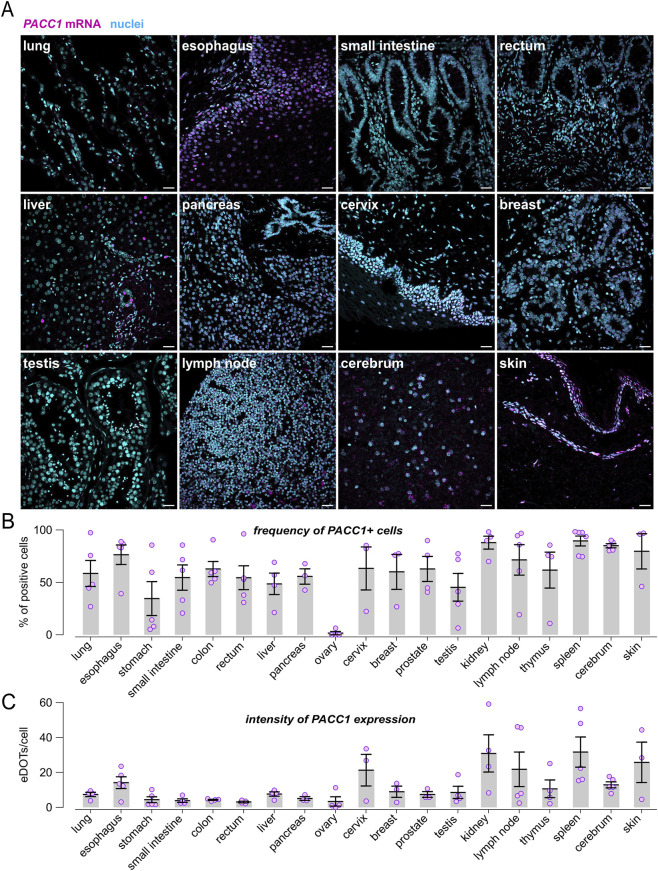
Expression profiling of *PACC1* in human tissues. RNAscope analysis of *PACC1* mRNA was performed on multi-organ human tissue microarrays. **(A)** Representative confocal images showing *PACC1* transcripts localization, visualized as magenta fluorescent dots (with each dot corresponding to a single mRNA molecule). Nuclei were stained in cyan. Scale bar: 25 μm. **(B)** Frequency of *PACC1*-positive cells across various human tissues. Cells containing one or more RNAscope signal dots were classified as *PACC1*-positive. **(C)** Quantification of *PACC1* mRNA abundance in *PACC1*-positive cells, measured as the number of RNAscope dots per cell. Data in **(B,C)** are presented as mean ± SEM (bars) with individual sample values indicated by dots. For each tissue type, 3-6 biological samples were analyzed (n = 3–6).

We applied RNAscope ISH technology to detect and visualize *PACC1* mRNA molecules across a panel of normal human tissues (Figure 1). For each tissue type, 3 to 6 samples comprising approximately 1,500 - 15,000 cells were examined by confocal microscopy combined with automated detection and quantification of RNAscope signals (fluorescent dots corresponding to individual *PACC1* mRNA molecules). Quantitative analysis revealed that *PACC1* transcripts are widely expressed across most tissues (Figure 1B). Specifically, the ovary showed a low proportion of *PACC1*-positive cells (1.58% ± 1.56%), whereas 30%–60% of cells expressed *PACC1* in the stomach, testis, liver, rectum, small intestine, pancreas, and lung. Intermediate expression levels (60%–80% positive cells) were observed in breast, thymus, colon, prostate, cervix, lymph node, esophagus, and skin, while the cerebrum, kidney, and spleen exhibited the highest expression, with 80%–90% of cells positive for *PACC1* (Figure 1B). Among *PACC1*-expressing cells, mRNA abundance, as indicated by the number of fluorescent dots per cell, varied by tissue type (Figure 1C). Lower expression levels (3–10 dots per positive cell) were observed in the ovary, stomach, pancreas, testis, colon, small intestine, breast, rectum, and liver. In contrast, higher expression levels (10–30 dots per positive cell) were detected in the thymus, lung, cerebrum, esophagus, cervix, lymph node, prostate, skin, kidney, and spleen. (Figure 1C). Notably, the brain emerged as one of the tissues with the highest *PACC1* expression. The cerebrum demonstrated both high frequency (84.99% ± 1.97% of *PACC1*-positive cells) and consistent expression intensity (12.94 ± 1.73 dots per positive cell) across all samples (Figures 1A–C).

### 3.2 PACC1 is expressed in both neuronal and glial cells

**FIGURE 2:**
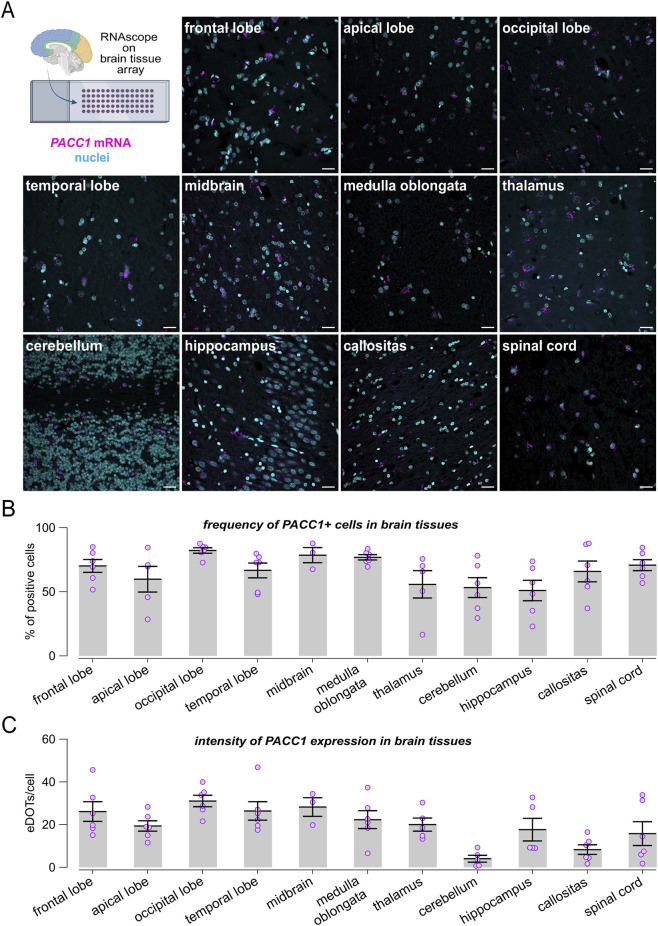
Expression profiling of *PACC1* in the human brain. RNAscope analysis of *PACC1* mRNA was performed on normal brain tissue microarrays. **(A)** Representative confocal images showing *PACC1* transcripts localization, visualized as magenta fluorescent dots, in a panel of human brain regions. Nuclei were stained in cyan. Scale bar: 25 μm. **(B)** Frequency of *PACC1*-positive cells across different brain regions. A cell was considered *PACC1*-positive if it contained one or more RNAscope signal dots. **(C)** Quantification of *PACC1* mRNA abundance in *PACC1*-positive cells, expressed as the number of RNAscope dots per cell. Data in **(B,C)** are presented as mean ± SEM (bars) with individual sample values indicated by dots. For each tissue type, 4-6 biological samples were analyzed (n = 4–6).

To comprehensively characterize *PACC1* expression in the brain, we extended the RNAscope analysis using a tissue microarray encompassing multiple normal brain regions (Figure 2A). *PACC1* was highly expressed across nearly all examined brain areas. The proportion of *PACC1*-positive cells ranged from approximately 50% in the hippocampus, cerebellum, thalamus, and apical lobe, to 65%–80% in the corpus callosum, temporal lobe, frontal lobe, spinal cord, medulla oblongata, midbrain, and occipital lobe (Figure 2B). Regional differences in *PACC1* mRNA abundance were also observed (Figure 2C). The cerebellum and corpus callosum exhibited the lowest expression levels, with 4.05 ± 1.61 and 8.33 ± 2.28 dots per positive cell, respectively, whereas other brain regions showed higher *PACC1* mRNA abundance, ranging from 15 to 30 dots per positive cell (Figure 2C).

**FIGURE 3:**
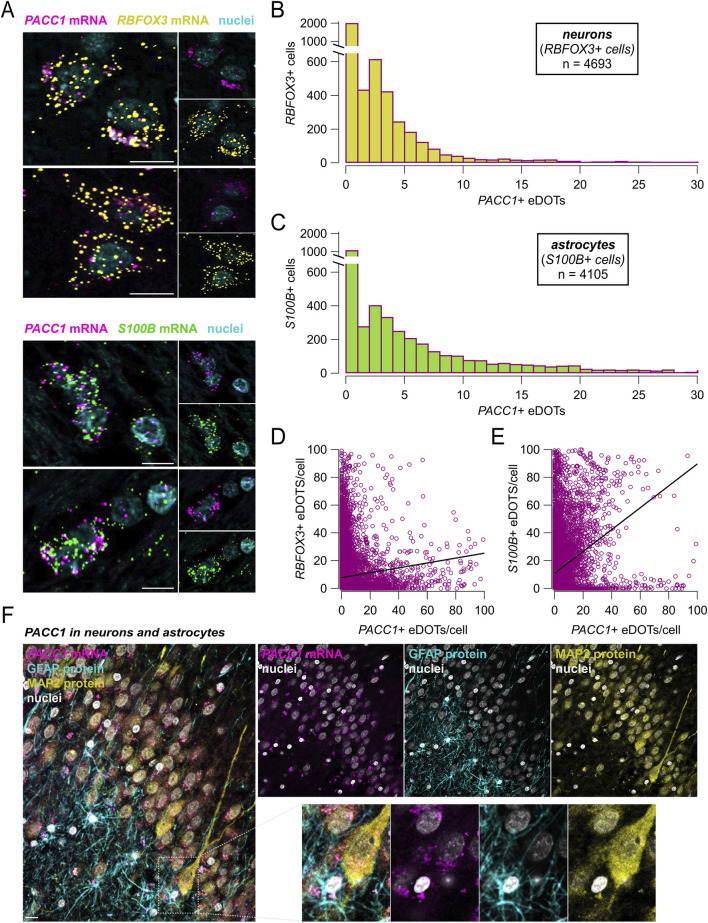
*PACC1* is expressed in both neurons and glia. **(A)** Representative RNAscope images showing co-expression of *PACC1* mRNA with either *RBFOX3* (neuronal marker) or *S100B* (astrocytic marker). *PACC1*, *RBFOX3*, and *S100B* transcripts are visualized as magenta, yellow, and green fluorescent puncta, respectively. Nuclei are counterstained in cyan. Scale bar: 10 μm. **(B–E)** Quantitative summary graphs showing the correlation between *PACC1* expression and *RBFOX3* **(B,D)** or *S100B* **(C,E)**. Data pooled from different multiple brain regions. **(F)** Representative images showing co-detection of *PACC1* mRNA (via RNAscope) and MAP2 and GFAP proteins (via immunofluorescence), markers of neurons and astrocytes, respectively. Scale bar: 10 μm. This analysis confirms *PACC1* expression in both cell types.

Next, we leveraged the multiplexing capability of RNAscope assays to evaluate *PACC1* expression in neuronal and glial cell populations. Co-expression of *PACC1* mRNA with *RBFOX3* and *S100B* transcripts, established markers for neurons and astrocytes, respectively, was visualized and quantified (Figure 3A). Cells exhibiting 10 or more fluorescent puncta for either the neuronal or glial marker were classified as neurons or astrocytes. Among 4,693 neurons and 4,105 astrocytes identified within brain tissue specimens, 53.91% of neurons and 69.52% of astrocytes expressed *PACC1* mRNA (Figures 3B,C). Notably, the intensity of *PACC1* expression differed between these cell types. In neurons, 87.51% of PACC1-positive cells exhibited low-to-moderate expression levels (1-9 dots), 11.11% showed high expression (10–30 dots), and 1.38% displayed very high expression (>30 dots) (Figure 3B). In contrast, astrocytes had a larger proportion of cells with higher *PACC1* expression: 70.21% in the low-to-moderate category, 26.77% with high expression, and 3.01% with very high expression (Figure 3C). Correlation analysis revealed a stronger positive association between *PACC1* and *S100B* expression than with *RBFOX3*, suggesting that *PACC1* is more abundantly expressed in astrocytes compared to neurons (Figures 3D,E). To further confirm these findings, we performed a combined detection of *PACC1* mRNA with MAP2 and GFAP proteins, as additional markers for neurons and astrocytes, respectively, using a multiplexed RNAscope ISH and immunofluorescence approach. This analysis confirmed the presence of *PACC1* mRNA in the cytoplasm and nuclei of both cell types (Figure 3F).

### 3.3 Tissue-specific expression of PACC1 splice isoforms

**FIGURE 4:**
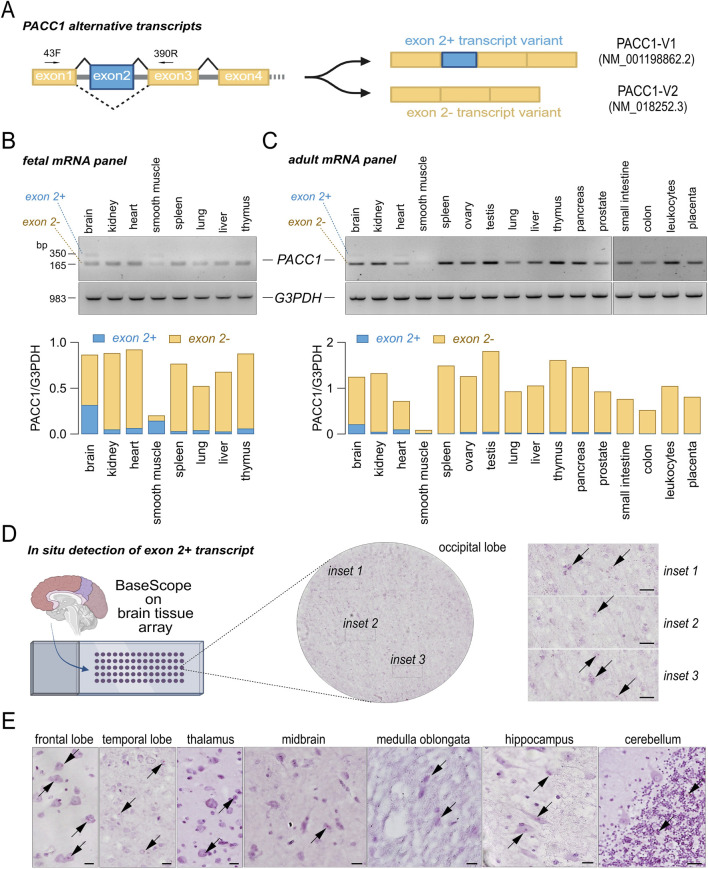
Tissue-specific expression of *PACC1* splice isoforms. **(A)** Schematic representation of the *PACC1* coding region, highlighting constitutive and alternative exons in yellow and blue boxes, respectively. Exon 2 is included in transcript variant 1 (PACC1-V1) and skipped in transcript variant 2 (PACC1-V2). The small black superimposed arrows indicate the position of the primers (43F and 390R) used for the RT-PCR. **(B,C)** Splicing patterns of *PACC1* exon 2 in human tissues. Panels of total RNAs from fetal **(B)** and adult **(C)** tissues were analyzed by RT-PCR using primers flanking exon 2. Top panels show representative agarose gel images with bands corresponding to exon 2-positive and exon 2-negative isoforms; bottom panels show amplification of the housekeeping gene *G3PDH* as a loading control. Transcript identities were confirmed by Sanger sequencing. RNA from adult tissues was pooled from 1 to 550 donors; RNA from fetal tissues was pooled from 13 to 59 donors. **(D)** Representative brightfield images showing expression and localization of PACC1-V1 in brain tissue sections. The whole-section overview (circular image) and higher-magnification insets (1-3) display red dots indicating PACC1-V1 mRNA (black arrows) detected by a 3ZZ BASEscope probe targeting 166-286 of NM_001198862.2. Scale bar: 20 μm. **(E)** Representative BASEscope images showing PACC1-V1 expression in various brain regions. Red puncta denote transcript localization. Scale bar: 20 μm.

**FIGURE 5:**
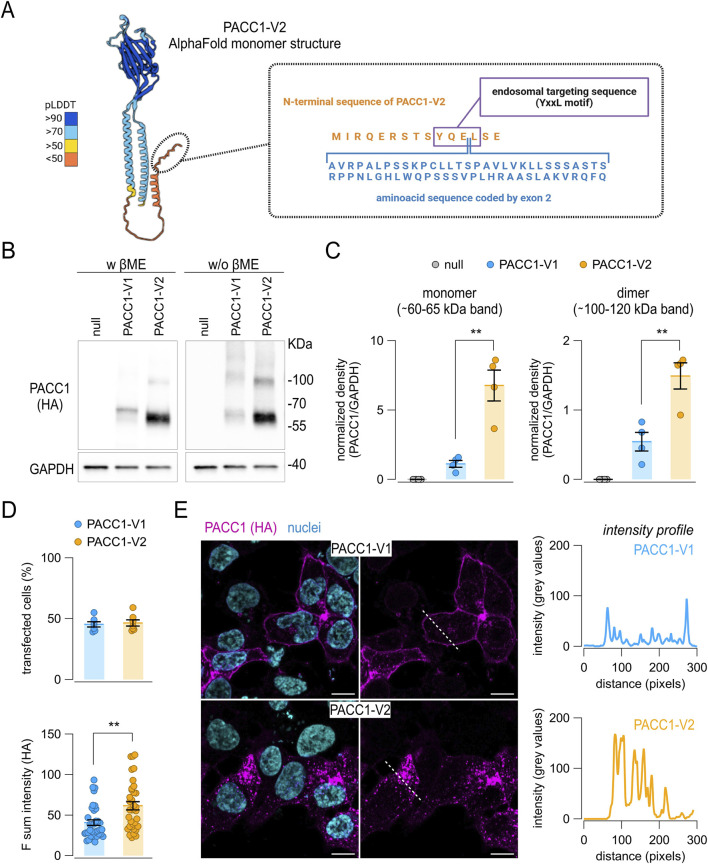
Expression of PACC1 splice isoforms *in vitro*. **(A)** Schematic representation of the human PACC1-V2 structure (AlphaFold code: AF-Q9H813-F1-v4), highlighting the position of the amino acid sequence encoded by the alternative exon 2 present in PACC1-V1. **(B,C)** Western blot analysis of PACC1 isoforms in total lysates from HEK-293 PACC1 KO cells transiently transfected with either PACC1-V1 or PACC1-V2. Isoforms were detected using an anti-HA antibody under denaturing (with β-mercaptoethanol, βME) and non-denaturing (without βME) conditions. GAPDH was used as a loading control (bottom images). Original Western blot images. **(C)** Densitometric analysis of Western blot bands showing quantification of monomeric (left panel) and dimeric (right panel) forms of PACC1. **, p < 0.01 by Student’s t-test (n = 4). **(D,E)** Immunofluorescence detection of PACC1 splice isoforms in transfected PACC1-KO cells. **(D)** Quantification of transfection efficiency (top) and fluorescence intensity (bottom) for PACC1-V1 and PACC1-V2. **, p < 0.01 by Student’s t-test (n = 6 independent transfections, each with 5 technical replicates). **(E)** Representative confocal images showing anti-HA immunostaining of PACC1 (magenta); nuclei were counterstained with DAPI (cyan). Scale bar: 5 μm. Intensity profiles of PACC1 staining along the dashed white lines in the images are shown in the adjacent graphs.

Analysis of ENSEMBL, UCSC, and GTEx databases for human *PACC1* splice variants identified two predominant isoforms that differ by the inclusion or skipping of exon 2 (Figures 4A, 5A). Variant 1 (accession no. NM_001198862.2), hereafter referred to as PACC1-V1, includes the exon 2 coding sequence and encodes a 411-amino acid protein. Variant 2 (accession no. NM_018252.3), referred to as PACC1-V2, lacks exon 2 and encodes a shorter, 350-amino acids protein. PACC1-V2 is considered the canonical isoform and has been the main focus of prior molecular and functional studies.

Using RT-PCR, we assessed the expression of *PACC1* transcript variants across a panel of normal fetal and adult human tissues (Figures 4B,C). PACC1-V2 was consistently expressed in nearly all tissues and developmental stages examined, except for adult smooth muscle, where its expression was notably low. In contrast, PACC1-V1 expression was largely restricted to fetal brain and smooth muscle, with lower levels detected in adult brain and heart (Figures 4B,C). To validate these findings, we performed *in situ* detection of PACC1-V1 in intact tissues using an isoform-specific BASEscope probe (Figure 4D). The brain was selected for this analysis as it showed the highest PACC1-V1 expression among both fetal and adult tissues. As shown in Figure 4, low-intensity signal (1-2 dots per cell) were observed in numerous cells across various adult brain regions, including the occipital, frontal, and temporal lobes, as well as the thalamus, medulla oblongata, hippocampus, and cerebellum (Figures 4D,E). In some regions, PACC1-V1 expression appeared cell-type specific. For example, in the cerebellum, PACC1-V1 was confined to cells of the granular layer, with no detectable signal in Purkinje cells or the molecular layer (Figure 4E).

### 3.4 Cellular expression of PACC1 splice isoforms

At the protein level, PACC1 splice isoforms differ by a 61-residues region encoded by exon 2, located near the N-terminus of the resulting protein. To explore the molecular consequences of exon 2 inclusion, we first attempted to generate a structural prediction model for PACC1-V1 using AlphaFold2. Since no experimental structures were available for human PACC1 containing exon 2, we selected the *Papio anubis* PACC1 ortholog as a modeling template (UniProt code: A0A2I3N0E6), owing to its conserved exon 2 region and high overall sequence identity (∼96%) with human PACC1-V1 (Supplementary Figure S2). Nonetheless, the entire N-terminal region of the predicted structure, also including that available for human PACC1-V2 (Figure 5A), remained unstructured and was assigned a very low per-residue confidence score (pLDDT <50), indicating limited structural reliability in this region (Supplementary Figure S3).

Since the exon 2-encoded region in PACC1-V1 affects a conserved endosomal targeting motif (YQEL), which has been implicated in PACC1 internalization, we performed a series of *in vitro* assays to assess the expression, subcellular localization, and functional properties of the two isoforms (Figure 5). For these experiments, we used HEK-293 cells in which endogenous *PACC1* was knocked out via CRISPR/Cas9-mediated genome editing (Supplementary Figure S4). The knockout cells were used to express a low level of either PACC1-V1 or PACC1-V2, each bearing a C-terminal triple HA epitope tag. Protein expression of the two PACC1 isoforms were first analyzed by Western blot under both reducing and non-reducing conditions to assess potential differences in protein stability or oligomerization state (Figures 5B,C). Total cell lysates revealed prominent bands at approximately 65 and 60 kDa for PACC1-V1 and PACC1-V2, respectively, corresponding to the monomeric forms; in addition, fainter bands were observed at higher molecular weights, consistent with dimeric and trimeric PACC1 forms (Figure 5B). Notably, PACC1-V1 expression was significantly lower than PACC1-V2, showing a ∼6-fold reduction in monomer levels and a ∼2.7-fold reduction in dimer levels (Figure 5C). Immunofluorescence analysis showed no significant difference in transfection efficiency between the two isoforms, with approximately 50% efficiency for both (Figure 5D). This analysis also confirmed the reduced expression of PACC1-V1, although the difference was less pronounced compared to Western blot results (Figure 5D). Notably, the subcellular localization patterns of the two isoforms were markedly distinct: PACC1-V1 was predominantly localized at the cell periphery, while PACC1-V2 displayed a wider cytoplasmic distribution with a characteristic punctate staining pattern (Figure 5E).

### 3.5 Intracellular trafficking of PACC1 splice isoforms

**FIGURE 6:**
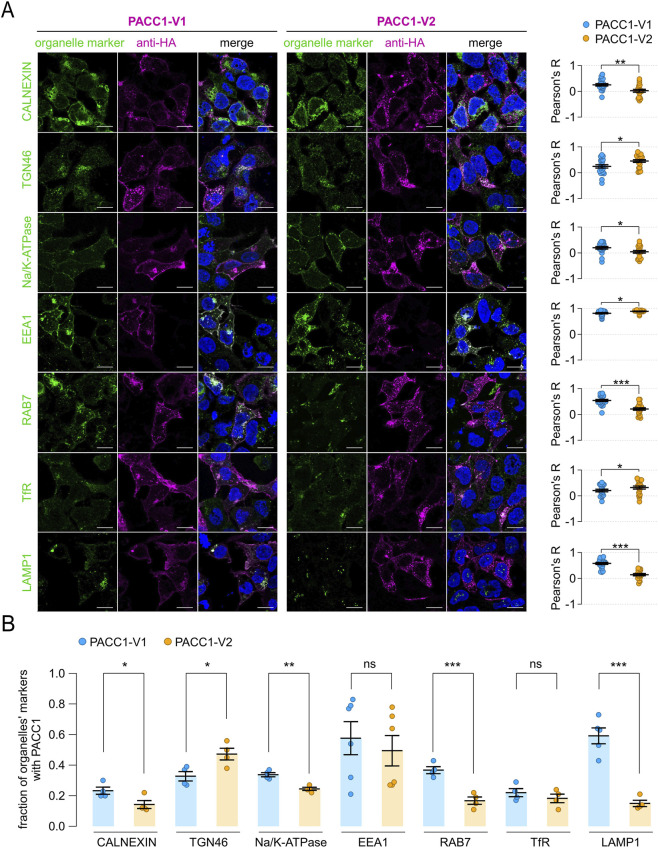
Subcellular localization of PACC1 splice isoforms. **(A)** Representative immunofluorescence images showing the localization of PACC1 isoforms, detected using an anti-HA antibody, in relation to various intracellular organelle markers: calnexin (endoplasmic reticulum), TGN46 (trans-Golgi network), Na^+^/K^+^-ATPase (plasma membrane), EEA1 (early endosomes), RAB7 (late endosomes), transferrin receptor (TfR, recycling endosomes), LAMP1 (lysosomes). Scale bar: 5 µm. Graphs on the right display quantitative co-localization analysis based on Pearson’s correlation coefficient (R), calculated from 15-25 cells across three independent transfections. *, p < 0.05; **p < 0.01, by Student’s t-test (n = 15–25). **(B)** Quantification of colocalization by high-content analysis of 150–500 cells from 4-6 independent transfection. Colocalization is expressed as the proportion of organelle marker area overlapping with the PACC1 signal. *, p < 0.05; **p < 0.01; ***, p < 0.001, by Student’s t-test (n = 4–6). These analyses highlight the differential distribution of PACC1-V1 and PACC1-V2 between plasma membrane and endosomal compartments, with quantitative metrics providing robust support for the observed localization patterns.

To deeply investigate the trafficking and subcellular localization of PACC1 isoforms, we performed co-immunostaining using several organelle-specific markers: calnexin (endoplasmic reticulum), TGN46 (trans-Golgi network), Na^+^/K^+^-ATPase (plasma membrane), EEA1 (early endosomes), RAB7 (late endosomes), transferrin receptor (TfR, recycling endosomes), and LAMP1 (lysosomes). Along the biosynthetic-secretory pathway, PACC1-V1 exhibited significantly higher localization to the endoplasmic reticulum and plasma membrane compared to PACC1-V2, which instead showed a modestly higher accumulation in the trans-Golgi network (Figures 6A,B). In the endocytic pathway, both isoforms displayed strong co-localization with the early endosomal marker EEA1, with Pearson correlation coefficients exceeding 0.8 (0.81 ± 0.03 for PACC1-V1 and 0.89 ± 0.013 for PACC1-V2; p < 0.05, Student’s t-test) (Figures 6A,B). However, in PACC1-V2-expressing cells, co-localization with EEA1 was predominantly observed in intracellular vesicles, whereas in PACC1-V1-expressing cells, a substantial fraction of the signal was detected at the cell periphery, consistent with its enhanced plasma membrane localization (Figure 6A). Additional differences emerged in later stages of the endocytic pathway: PACC1-V1 showed significantly higher co-localization with late endosomes (RAB7) and lysosomes (LAMP1), while PACC1-V2 exhibited greater co-localization with recycling endosomes (TfR) (Figures 6A,B). Taken together, these results indicate that exon 2 inclusion shifts, rather than abolishes, endosomal targeting of PACC1, leading to altered compartmental distribution and trafficking dynamics of PACC1-V1 isoform.

### 3.6 Function of PACC1 splice isoforms

Based on their distinct intracellular localization patterns, we hypothesize that PACC1 splice isoforms may differentially contribute to acid-activated chloride transport within endosomes and across the plasma membrane, two known sites of PACC1 activity. Indeed, in endosomes, PACC1 channel activity mediates chloride efflux from the lumen, acting as brake to prevent hyper acidification, while at the plasma membrane PACC1-mediated chloride influx has been associated with acidotoxicity.

**FIGURE 7:**
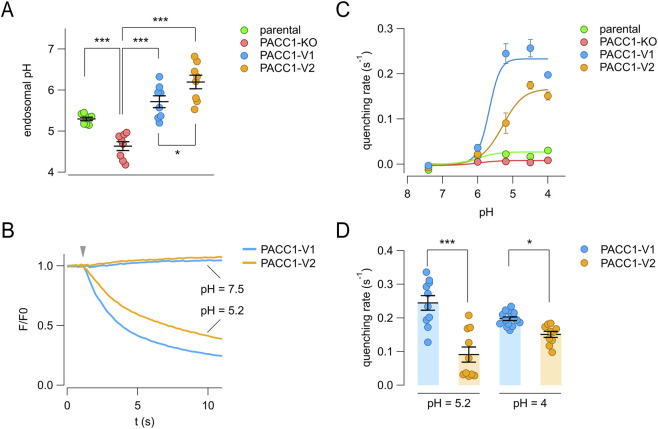
Functional characterization of PACC1 splice isoforms. **(A)** Endosomal pH was measured using a ratiometric flow cytometry assay with dual transferrin probes in parental HEK-293 cells, PACC1-KO cells, and PACC1-KO cells transfected with either PACC1-V1 or PACC1-V2. The graph shows mean ± SEM from 10,000 cells per replicate (n = 8). *, p < 0.05; ***, p < 0.001 by one-way ANOVA (followed by Tukey’s *post hoc* test). **(B–D)** Chloride channel activity at the plasma membrane was assessed using the halide-sensitive YFP (HS-YFP) assay. **(B)** Representative traces showing HS-YFP fluorescence quenching upon iodide addition (arrowhead) at extracellular pH 7.5 or 5.2. **(C)** Quenching rates (reflecting plasma membrane channel activity) at various extracellular pH values. Data are shown as mean ± SEM (n = 10–15). Curves were fitted using the Hill equation. **(D)** Comparison of maximal quenching rates for PACC1-V1 and PACC1-V2 at the indicated pH. *, p < 0.05; ***, p < 0.001 by Student’s t-test (n = 10–15).

To test this hypothesis, we quantified endosomal pH using a ratiometric method based on cell loading with fluorescent pH-insensitive (AlexaFluor-633-conjugated) and pH-sensitive (FITC-conjugated) transferrin probes. Their fluorescence ratios were measured by flow cytometry and converted to pH values using standard calibration buffers. For these experiments, PACC1 splice isoforms were tagged with mCherry (instead of triple HA epitope) to allow gating of transfected cells during flow cytometry analysis. As expected, PACC1-KO cells exhibited a significantly more acidic endosomal pH compared to parental wild-type cells (endosomal pH was 5.30 ± 0.04 for parental cells and 4.63 ± 0.11 for KO cells) (Figure 7A). Overexpression of either PACC1 isoforms led to endosomal alkalinization relative to both KO and parental cells, with PACC1-V2 producing significantly higher pH values than PACC1-V1 (5.68 ± 0.16 for PACC1-V1 and 5.85 ± 0.23 for PACC1-V2) (Figure 7A).

We next assessed the plasma membrane activity of the PACC1 isoforms using the halide-sensitive YFP (HS-YFP) assay, in which the rate of YFP fluorescence quenching reflects chloride channel activity. KO cells were co-transfected with HS-YFP and either PACC1-V1 or PACC1-V2 tagged with mCherry. As shown in Figure 7B, no quenching was observed at neutral extracellular pH (7.5), whereas acidification to pH 5.2 triggered a rapid quenching response, with an apparently higher rate in cells expressing PACC1-V1 (Figure 7B). Analysis of the pH-dependence of PACC1 channel activity revealed similar pH sensitivity between the two isoforms, with half-maximal activation at pH 5.53 ± 0.33 for PACC1-V1 and pH 5.31 ± 0.23 for PACC1-V2 (Figure 7C). However, the maximal activity was significantly higher for PACC1-V1 compared to PACC1-V2 (0.23 ± 0.03 vs. 0.17 ± 0.02, respectively), probably reflecting the larger presence of PACC1-V1 at the plasma membrane (Figures 7C,D). Collectively, these results indicate that alternative splicing of PACC1 produces functional channels with higher presence and activity in endosomes (PACC1-V2) or at the plasma membrane (PACC1-V1), depending on the exclusion or inclusion of exon 2.

### 3.7 PACC1-V1 isoform confers increased cellular susceptibility to acid-induced cell death

**FIGURE 8:**
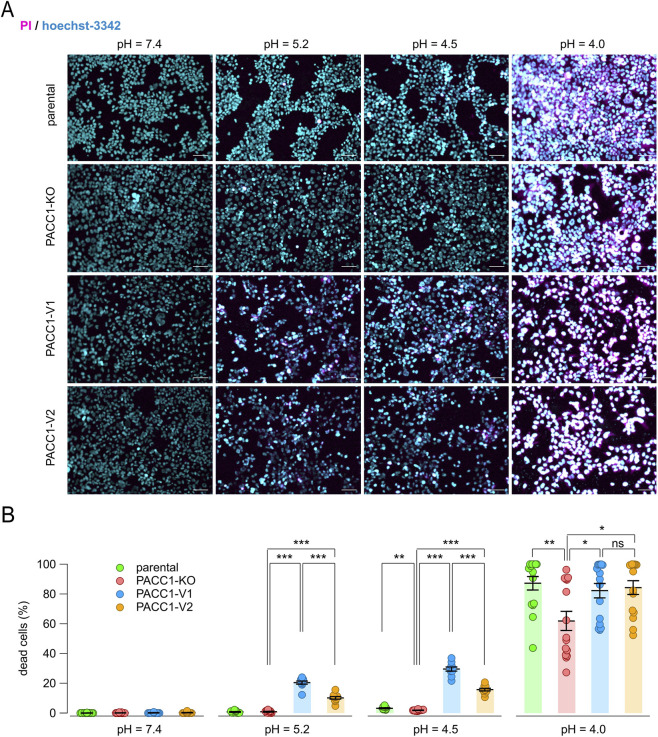
Contribution of PACC1 splice isoforms to acid-induced cell death. **(A)** Representative images from the acid-induced cell death assay performed on parental, PACC1-KO, PACC1-V1, and PACC1-V2 HEK-293 cells at different extracellular pH (7.4, 5.2, 4.5, and 4.0). Cells were stained with Hoechst 33342 (blue) and propidium iodide (PI, red). Scale bar: 30 µm. **(B)** Summary graph showing the percentage of dead cells in the different samples and conditions. *, p < 0.05; **, p < 0.01; ***, p < 0.001 with 1-way ANOVA (followed by Tukey’s *post hoc* test); n = 10–15.

Finally, we evaluated the contribution of PACC1 isoforms to acid-induced cell death by exposing cells to acidic solutions and performing dual staining with propidium iodide (to label dead cells) and Hoechst-33342 (to label all nuclei) (Figure 8). As expected, PACC1 KO cells were more resistant to acid-induced cell death, even at highly acidic pH values (4.5–4.0). In contrast, overexpression of either PACC1 isoform increased susceptibility to acidotoxicity, as indicated by a higher proportion of propidium iodide-positive cells at extracellular pH 5.2 and 4.5 (Figures 8A,B). Notably, the extent of acid-induced cell death was significantly increased in cells expressing PACC1-V1 compared to those expressing PACC1-V2, particularly at pH 5.2 and 4.5, conditions under which parental cells typically exhibit resistance (Figure 8A).

## 4 Discussion

In this study, we provide a comprehensive characterization of *PACC1* expression and splice isoform diversity across human tissues, with a particular focus on the brain. Utilizing RNAscope ISH technology, we showed that *PACC1* mRNA is broadly expressed across diverse normal human tissues, with notable heterogeneity in both the proportion of positive cells and transcript abundance. Among all tissues analyzed, the cerebrum and other brain regions exhibited particularly high and uniform expression, underscoring the potential importance of *PACC1* in central nervous system physiology.

Previous studies in rodents have shown that *PACC1*-encoded channel is activated by extracellular acidification and plays a critical role in mediating acid-induced neuronal cell death, a process implicated in ischemia, cancer, and inflammation (Ullrich et al., 2019; Yang et al., 2019; Osei-Owusu et al., 2022). Notably, genetic ablation of *Pacc1* in mice attenuated brain damage following ischemic stroke, identifying *PACC1* as a potential therapeutic target for limiting ischemia-induced neurotoxicity (Yang et al., 2019).

Here, we expand upon this knowledge by demonstrating that *PACC1* is expressed in both human neurons and astrocytes. Quantitative analyses revealed that astrocytes express higher levels of *PACC1* compared to neurons, as evidenced by co-localization and correlation analyses using astrocytic (*S100B*, *GFAP*) and neuronal (*RBFOX3*, *MAP2*) markers at both transcript and protein levels. Given the essential roles of astrocytes in maintaining brain homeostasis, synaptic function, and neuroprotection, the preferential expression of *PACC1* in astrocytes suggests potential involvement in the regulation of ionic balance and pH buffering, warranting further functional investigation (Sidoryk-Wegrzynowicz et al., 2011; Liu and Chopp, 2016; Becerra-Calixto and Cardona-Gómez, 2017; Collyer and Blanco-Suarez, 2023).

A key contribution of this study is the detailed characterization of two major *PACC1* splice isoforms, PACC1-V1 and PACC1-V2, which differ by the inclusion of exon 2. Alternative splicing is a fundamental mechanism of proteomic diversification, particularly in the brain, where it regulates neurodevelopment, cell-type identity, and synaptic function (Raj et al., 2014; Raj and Blencowe, 2015; Vuong et al., 2016; Furlanis and Scheiffele, 2018). Consistent with this paradigm, we found that the canonical isoform, PACC1-V2, is broadly expressed across tissues, whereas PACC1-V1 shows a more restricted pattern, predominantly in fetal brain and smooth muscle. This tissue-specific distribution suggests that the two isoforms may fulfill distinct physiological and developmental roles, possibly reflecting specialized demands for acid-induced channel activity. Furthermore, the selective expression of PACC1-V1 in fetal tissues raises the possibility that splicing misregulation could contribute to developmental brain disorders (Vuong et al., 2016; Olmos et al., 2024).

Inclusion of exon 2 in PACC1-V1 disrupts a conserved endosomal targeting motif (Osei-Owusu et al., 2021; Ko et al., 2024), a molecular alteration that likely underlies the observed differences in subcellular localization and trafficking. Functional assays in heterologous human cell systems revealed that PACC1-V1 and PACC1-V2 display distinct intracellular distributions. PACC1-V1 predominantly localizes to the plasma membrane, lysosomes, and endoplasmic reticulum, while PACC1-V2 is mainly associated with the trans-Golgi network and endosomes. This spatial segregation aligns with their observed functional profiles: PACC1-V2 more effectively alkalinizes endosomal compartments, supporting a role in regulating organellar pH (Osei-Owusu et al., 2021), while PACC1-V1 shows higher activity under acidic extracellular conditions, consistent with enhanced plasma membrane function (Ko et al., 2024).

We note that the absolute endosomal pH values measured with the transferrin-based ratiometric assay in this study are lower than canonical early/recycling endosome values. However, the assay provides a reliable relative comparison between genotypes, which is the primary basis for our conclusions. Plasma membrane chloride transport was evaluated using HS-YFP as a functional proxy, allowing isoform-specific comparisons under identical experimental conditions. While patch-clamp recordings would offer detailed biophysical characterization at the plasma membrane, they cannot readily capture compartment-specific function in endosomes, which is central to our study. Furthermore, all isoforms were analyzed using identically tagged constructs, minimizing potential artifacts from the C-terminal 3×HA/mCherry tags. Collectively, these considerations define the scope of our functional assays while supporting the robustness of the observed isoform-specific effects.

Moreover, cells expressing PACC1-V1 were more susceptible to acid-induced cell death, supporting a role in mediating acidotoxicity under pathological or developmental conditions marked by extracellular acidosis (Wemmie et al., 2013; Ullrich et al., 2019; Yang et al., 2019; Osei-Owusu et al., 2022). In contrast, PACC1-V2 may play a protective role by maintaining endosomal pH homeostasis (Osei-Owusu et al., 2021; Zeziulia et al., 2022). Together, these findings illustrate how alternative splicing modulates PACC1’s intracellular trafficking and function, thereby fine-tuning its physiological and pathological roles.

PACC1 is increasingly recognized as a multifunctional proton-activated chloride channel implicated in a wide spectrum of disease processes across different organ systems. In a murine model of lumbar spine instability, PACC1 was upregulated in osteoclasts and promoted acid-activated chloride conductance that enhanced osteoclast fusion and endplate bone resorption, ultimately leading to increased porosity and pain (Xue et al., 2024). Genetic knockout of *Pacc1* significantly attenuated these degenerative changes and pain behaviors without disrupting normal bone homeostasis, highlighting a potential for targeted intervention in acid-driven bone disorders such as osteoarthritis, ankylosing spondylitis, rheumatoid arthritis, heterotopic ossification, and enthesopathy (Xue et al., 2024). Notably, *PACC1* has also been identified within the ataxin-1 interaction network, implicating it in the pathogenesis of spinocerebellar ataxia type 1 (SCA1), a neurodegenerative disorder marked by protein aggregation and neuronal dysfunction (Chen et al., 2022). Additionally, genetic evidence from a study of a consanguineous Pakistani family with autosomal recessive intellectual disability identified RDH14 as a novel candidate gene and proposed functional links to *PACC1*, suggesting that disruption of chloride homeostasis or downstream signaling pathways may contribute to cognitive impairment (Pastore et al., 2021). Collectively, these findings position *PACC1* as a critical regulator of acid-sensing chloride transport with emerging roles in bone pain and central nervous system function and support its candidacy as a therapeutic target in both somatic and neurodevelopmental disorders.

In summary, our findings broaden the understanding of *PACC1* biology, linking splice isoform diversity to cellular localization and functional specialization. Future studies should elucidate the mechanisms regulating *PACC1* splicing and investigate isoform-specific roles in neural and non-neural tissues. In addition, defining the molecular interactions that govern isoform-specific trafficking and channel regulation will be critical for developing targeted strategies. Given its disease-selective expression and diverse roles, modulating PACC1 activity or splicing may offer novel avenues for intervention in acid-associated disorders such as ischemia, neurodegeneration, and skeletal pathologies.

### 4.1 Conclusion

This study expands our understanding of *PACC1* by providing a comprehensive analysis of its tissue distribution, splice isoform diversity, and cell-type specificity in the human brain. We show that alternative splicing generates functionally distinct isoforms with divergent subcellular localization and compartment-specific roles, influencing both plasma membrane chloride transport and endosomal pH regulation. These findings highlight a mechanism by which PACC1 activity may be fine-tuned in different physiological and pathological contexts. The preferential expression of PACC1 in astrocytes, along with its involvement in acidotoxicity, neurodevelopment, and systemic disorders such as bone degeneration and intellectual disability, underscores its broad biological relevance. We note that functional assays, including HS-YFP and endosomal pH measurements, provide robust relative comparisons between isoforms, although further biophysical and mutational studies could refine the mechanistic understanding.

Collectively, our findings position PACC1 as a key regulator of acid-sensing chloride signaling and suggest that targeting its isoform-specific functions or splicing regulation may offer novel therapeutic opportunities in both neurodegenerative and musculoskeletal diseases.

## Data Availability

The original contributions presented in the study are included in the article/Supplementary Material, further inquiries can be directed to the corresponding author.
