# Supplementary material for: Alternative splicing regulates PACC1 function and promotes acidosis-induced cytotoxicity
Source: Front Cell Dev Biol. 2026 Jan 30;13:1754079. doi: 10.3389/fcell.2025.1754079 (PMC12901492; doi:10.3389/fcell.2025.1754079)

Original Western blot images for panel B of Fig. 5

With βME

Anti-HA antibody
(PACC1)

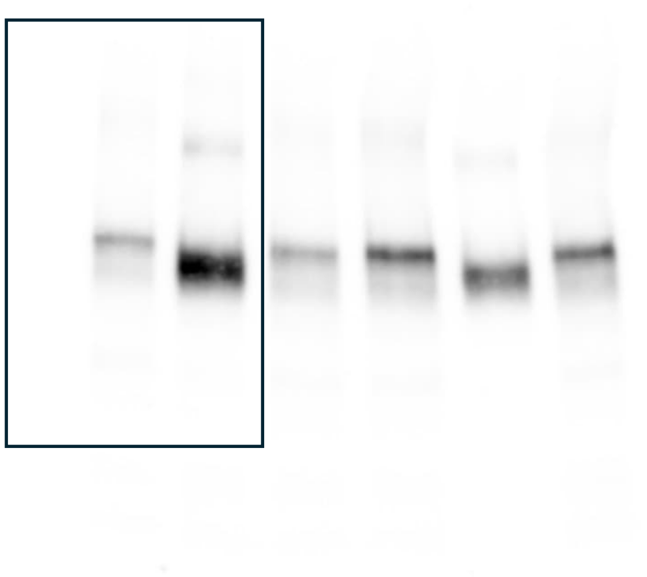

Original Western blot images for panel B of Fig. 5

With βME

Anti-GAPDH

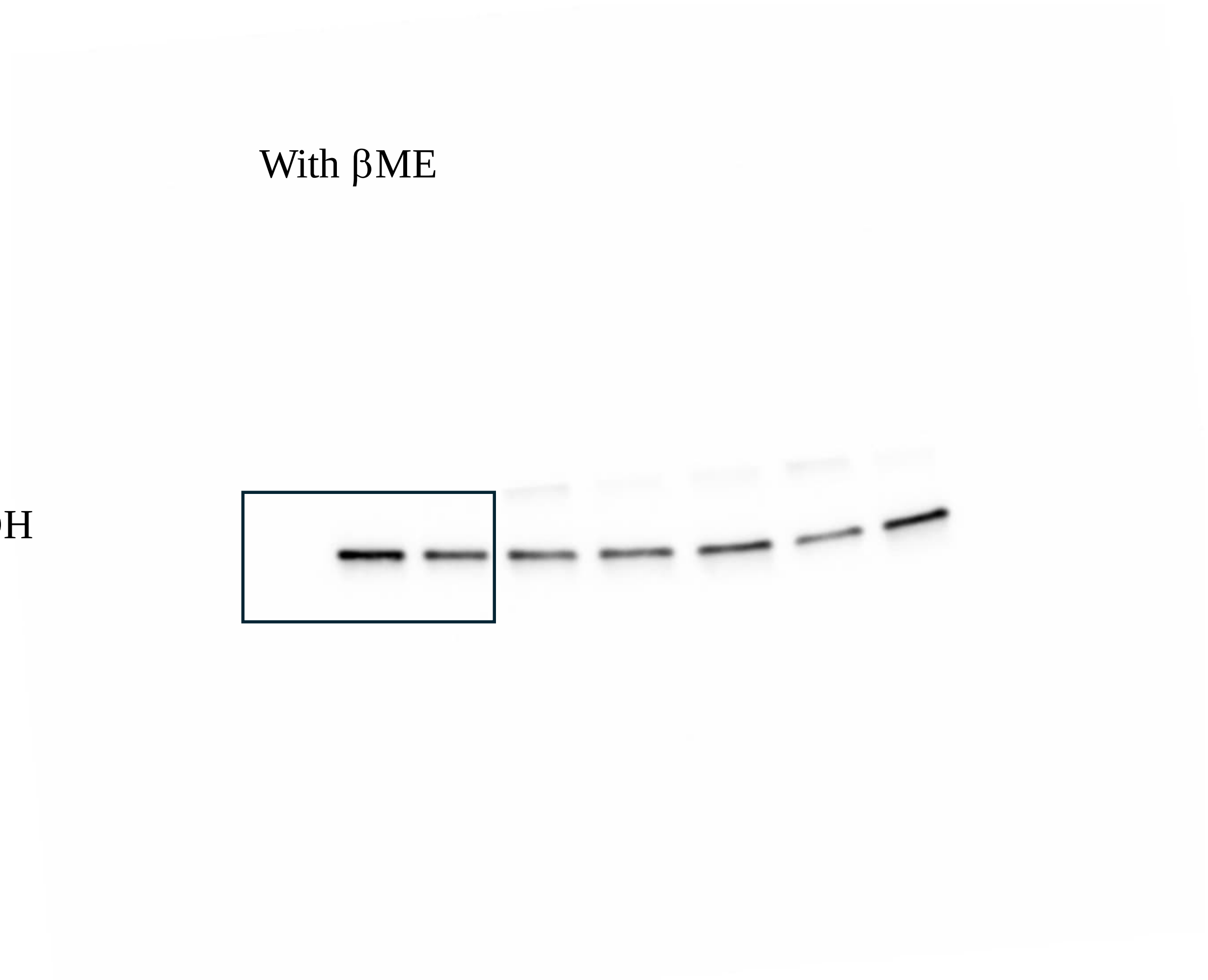

Original Western blot images for panel B of Fig. 5

w/o βME

Anti-HA antibody
(PACC1)

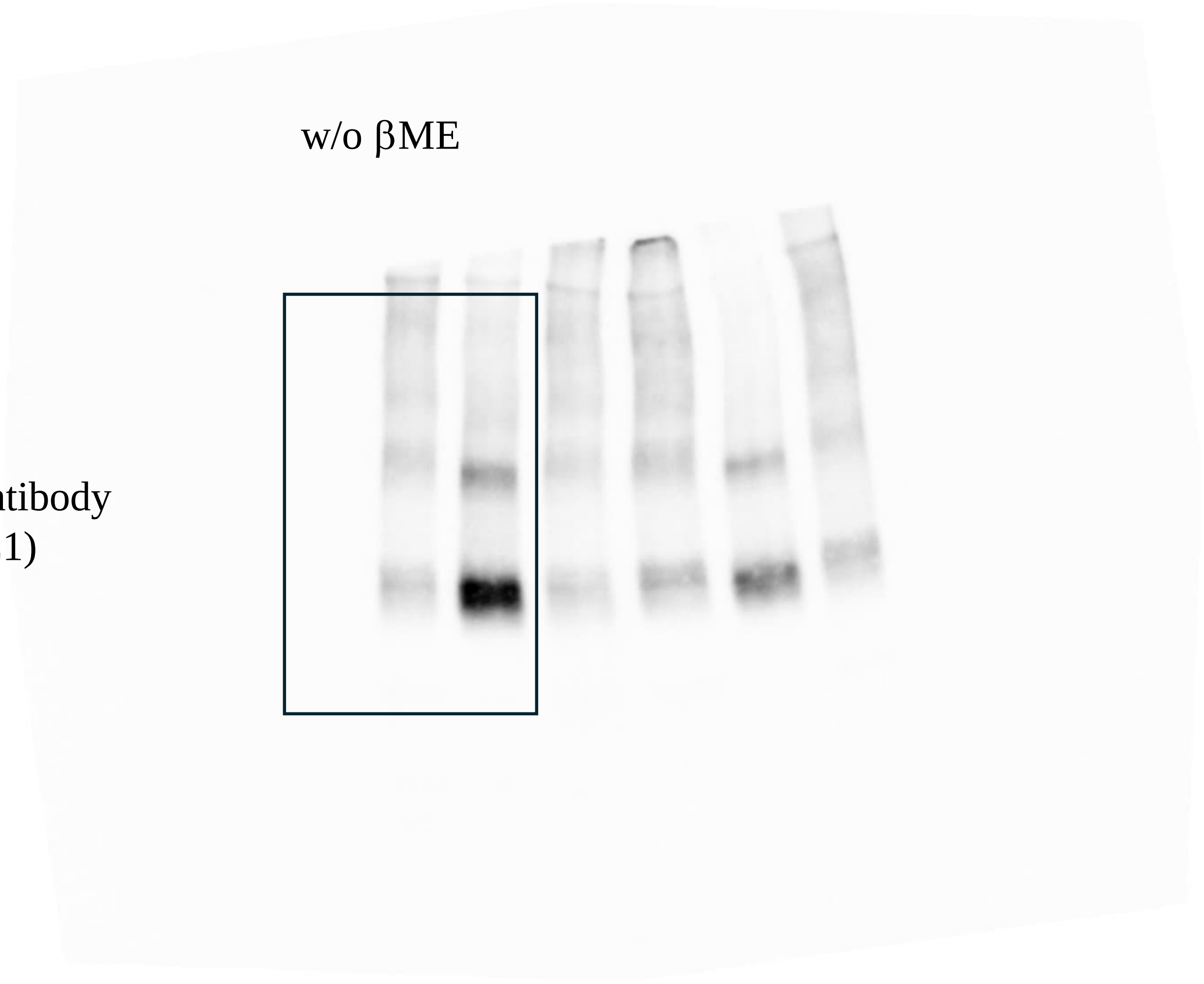

Original Western blot images for panel B of Fig. 5

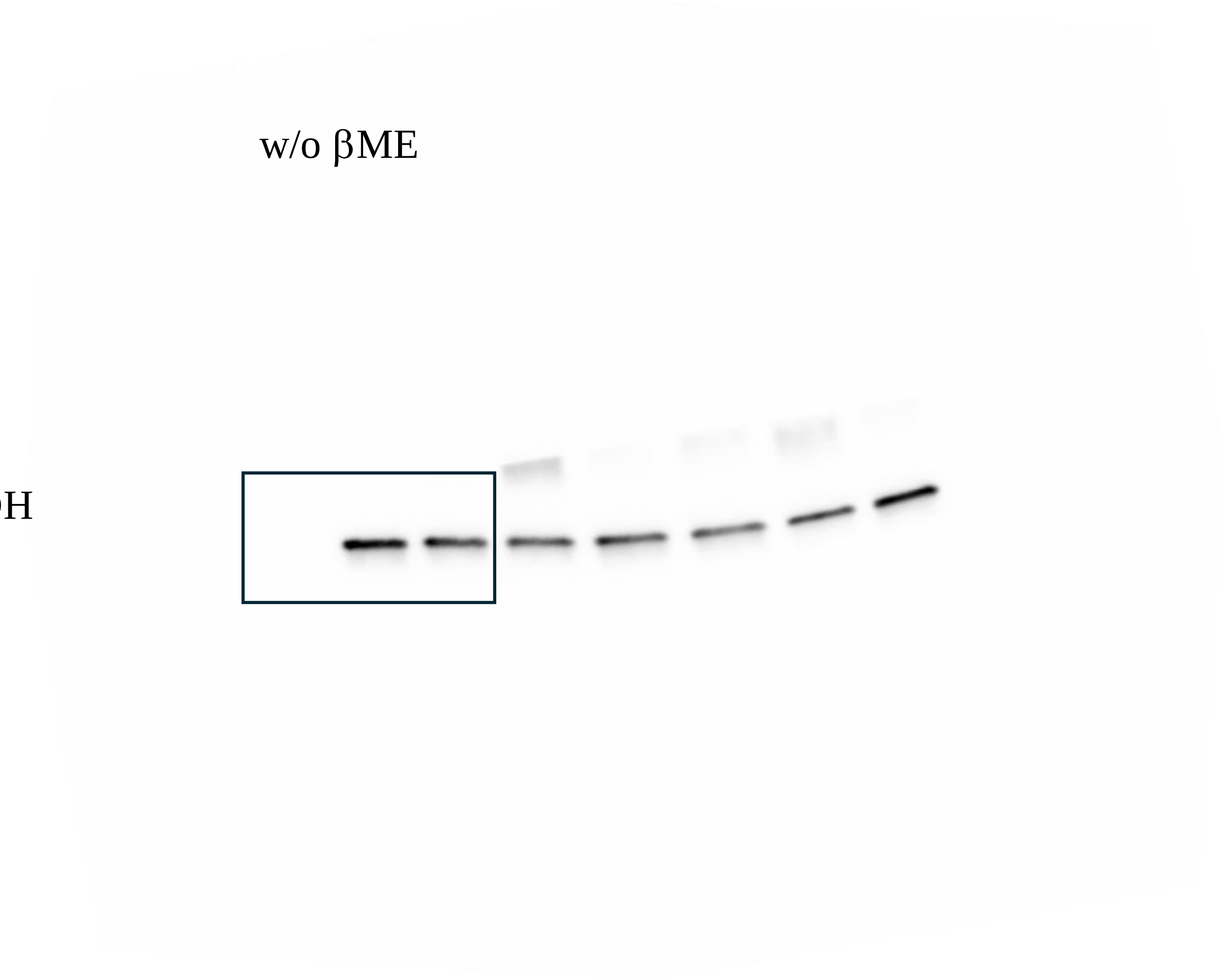

Supplement: Supplementary file 1 [file Supplementaryfile1.pdf]
