# Supplementary material for: Alternative splicing regulates PACC1 function and promotes acidosis-induced cytotoxicity
Source: Front Cell Dev Biol. 2026 Jan 30;13:1754079. doi: 10.3389/fcell.2025.1754079 (PMC12901492; doi:10.3389/fcell.2025.1754079)
Supplement: Supplementary file 2 [file DataSheet1.docx]

Supplementary Material

Alternative Splicing Regulates PACC1 Function and Promotes Acidosis-Induced Cytotoxicity

**Serena Tamburro^1^, Giulia Gorrieri^1,2^, Niccolò Callegari^1^, Floriana Guida^1^, Francesca Antonini^3^, Ilaria Musante^2^, Simona Baldassari^2^, Federico Zara^1,2^, Paolo Scudieri^1,2,*^**

^1^ Department of Neurosciences, Rehabilitation, Ophthalmology, Genetics, Maternal and Child Health (DiNOGMI), University of Genoa, Genoa, Italy

^2^ Medical Genetics Unit, IRCCS Istituto Giannina Gaslini, Genoa, Italy

^3^ Core facilities for Omics Science, IRCCS Istituto Giannina Gaslini, Genoa, Italy

*** Correspondence:**Paolo Scudieri, [paolo.scudieri@unige.it](mailto:paolo.scudieri@unige.it)

# Supplementary Figures


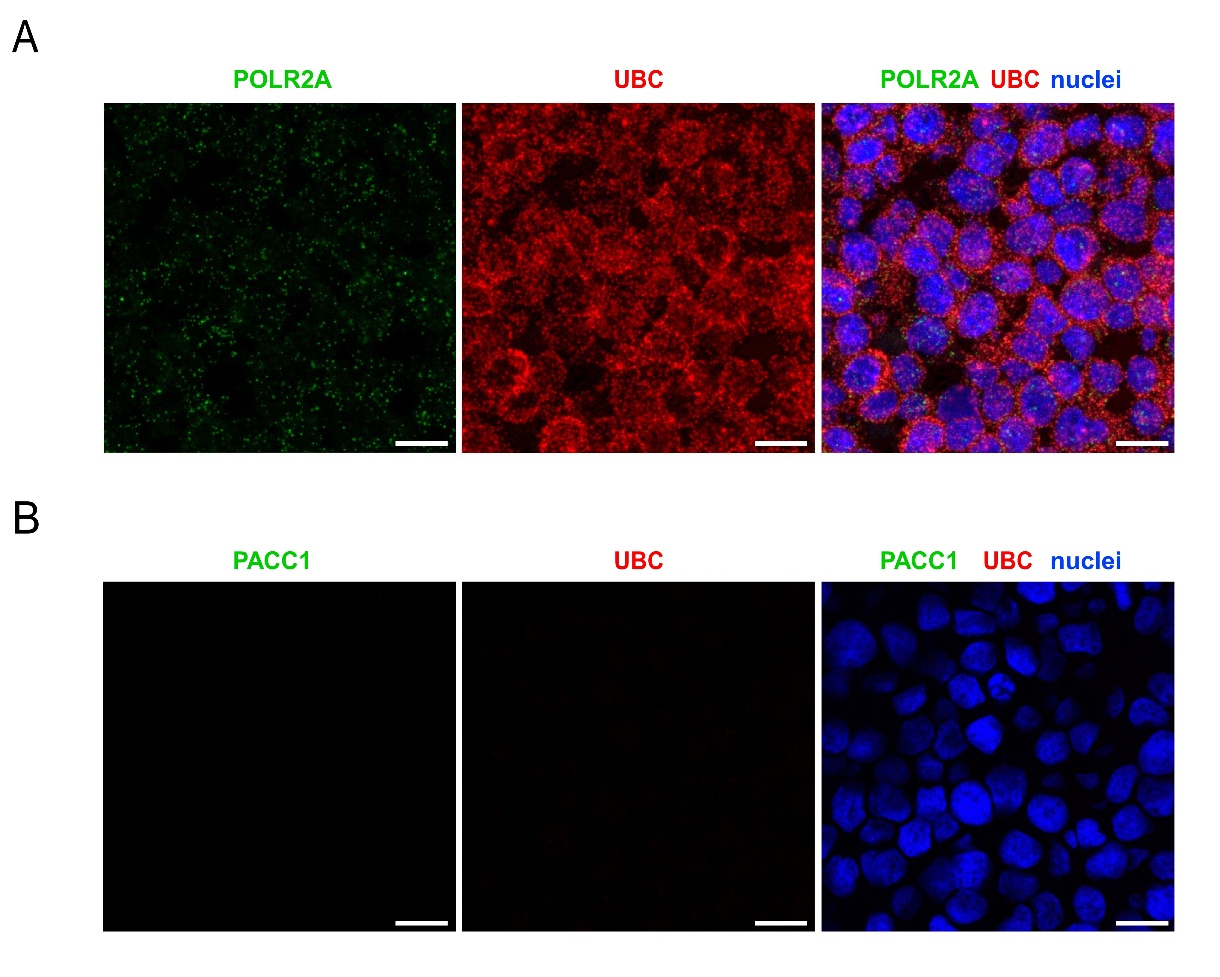


**Supplementary Figure S1. Validation of RNAscope probe specificity.** Representative RNAscope images showing specificity controls for PACC1 detection. (**A**) Positive control probes targeting housekeeping genes with low (POLR2A) and high (UBC) expression produced the expected punctate signal, confirming RNA integrity and assay performance. (**B**) RNase A pretreatment abolished PACC1 and UBC RNAscope signal confirming probe specificity. Nuclei are counterstained with DAPI. Scale bars, 10 µm.


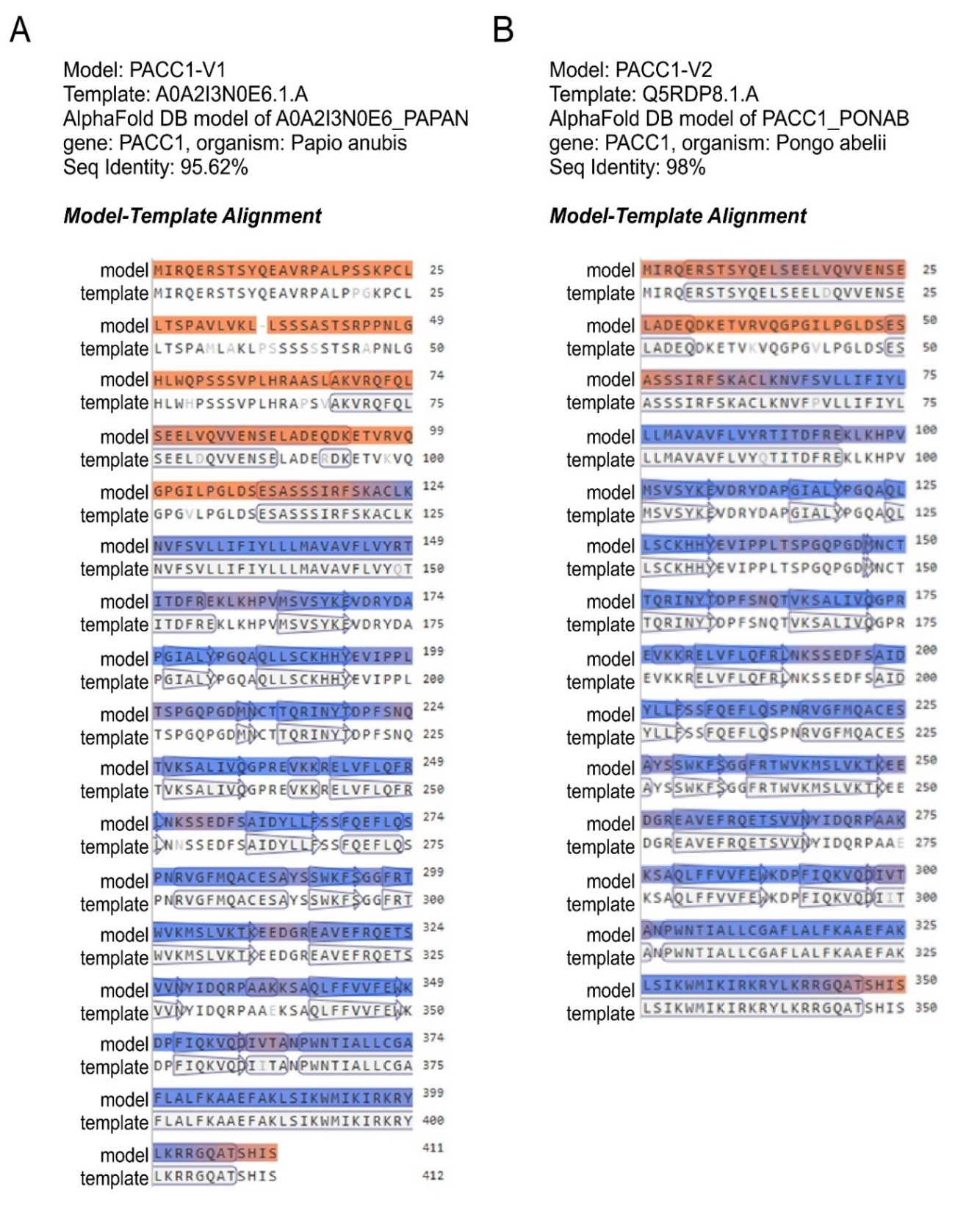


**Supplementary Figure S2. Structural modeling PACC1 splice isoforms.** Sequence alignment between the modeled structures of PACC1-V1 (left) and PACC1-V2 (right) and their corresponding template (Papio anubis PACC1 ortholog A0A2I3N0E6.1.A for PACC1-V1; Pongo abelii PACC1 ortholog Q5RDP8.1.A for PACC1-V2). The alignment illustrates sequence conservation and structural divergence between the two isoforms.


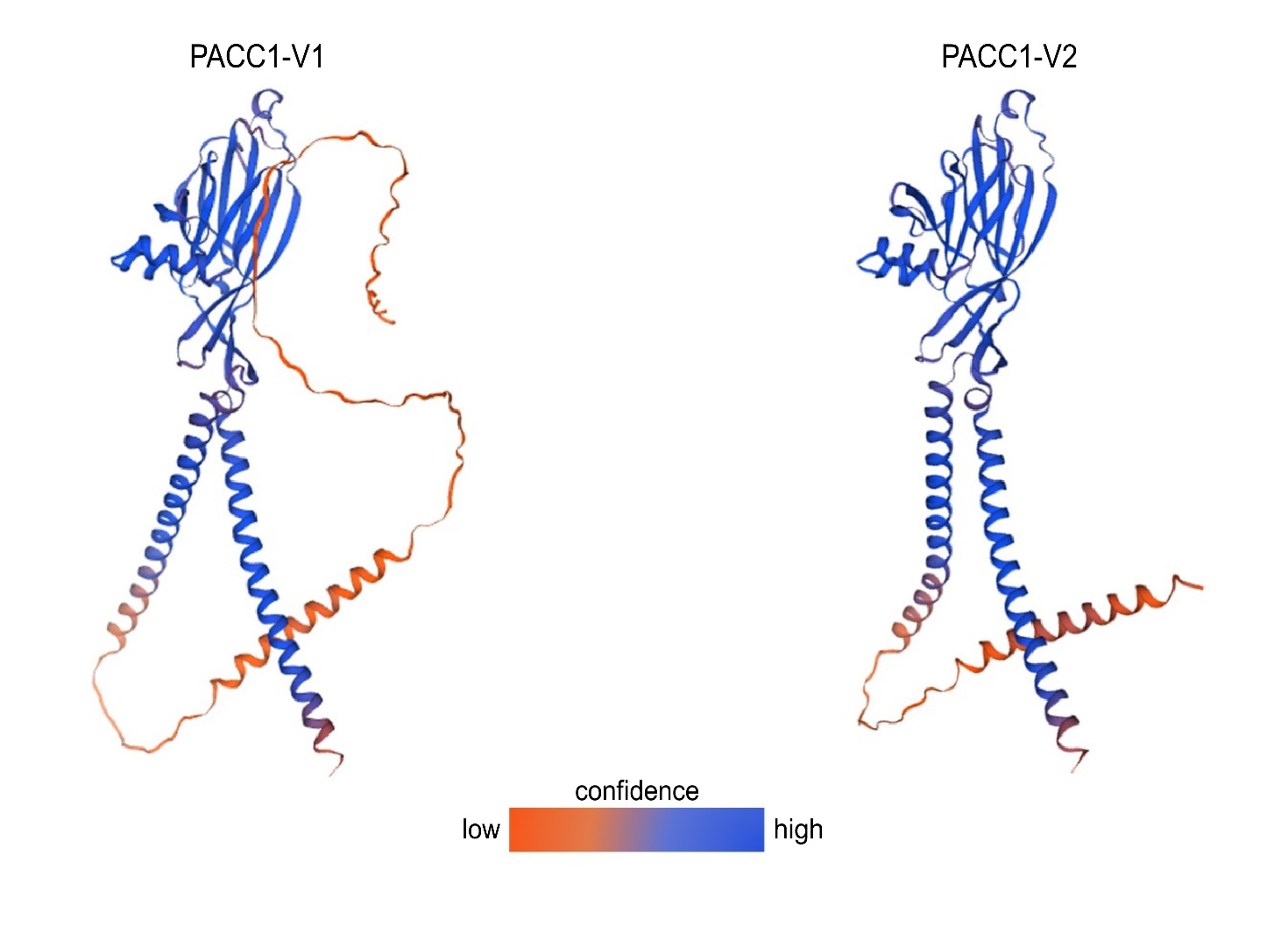


**Supplementary Figure S3. Structural prediction models of PACC1 splice isoforms.** Schematic representations of the predicted structures of PACC1-V1 (left) and PACC1-V2 (right) generated using AlphaFold. Models are color-coded based on per-residue confidence scores (pLDDT), ranging from orange (low confidence) to blue (high confidence). The region encoded by exon 2, present only in PACC1-V1, contributes to low-confidence predictions in the N-terminal domain.


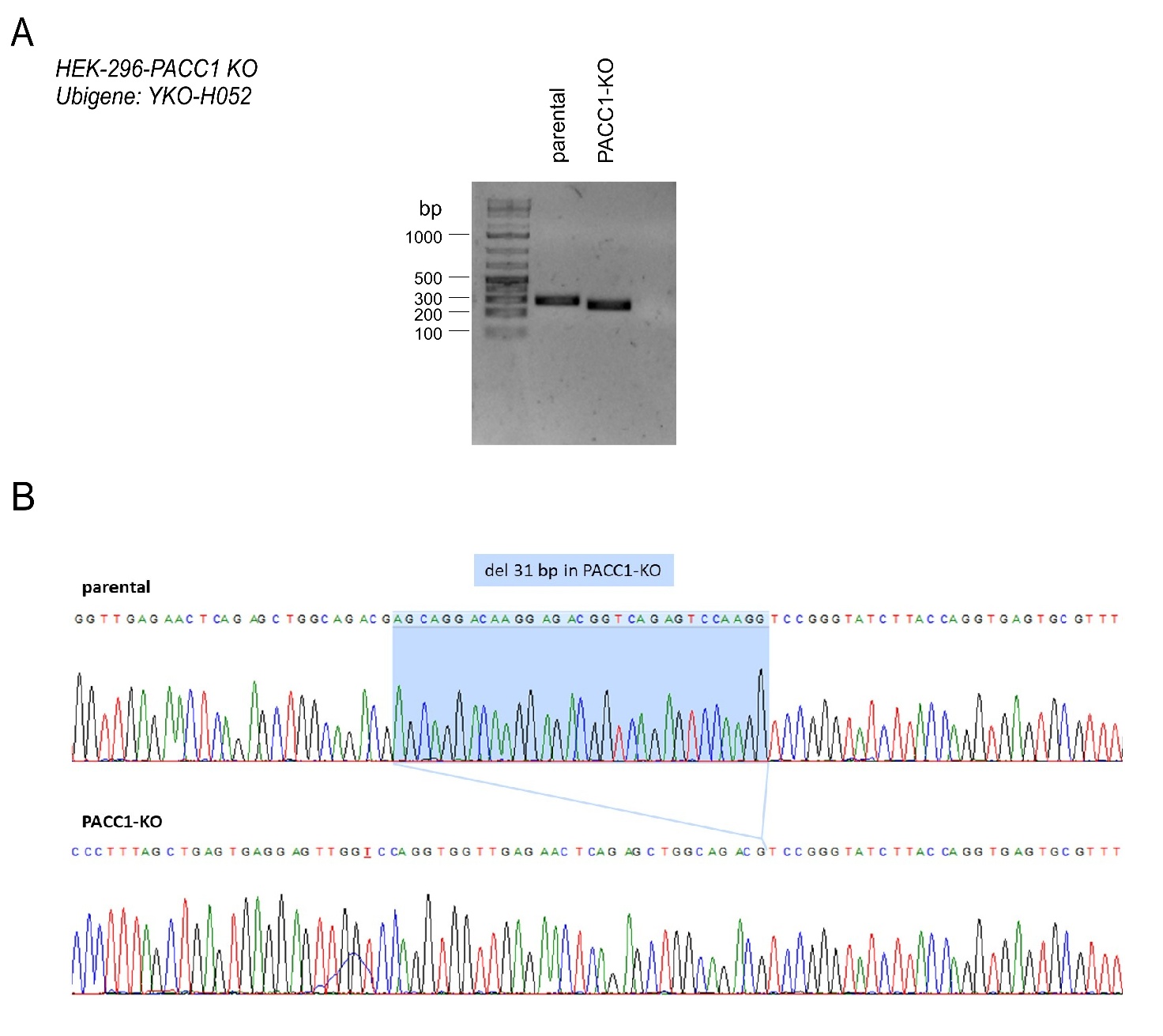


**Supplementary Figure S4. Validation of PACC1 knockout (KO) in HEK-293 cells.** HEK-293 PACC1 KO cells obtained from Ubigene (catalog # YKO-H052) were validated using PCR and Sanger sequencing. (A) Agarose gel showing PCR amplification using primers flanking the deleted region in exon 3 of the *PACC1* coding sequence. (B) Sanger sequencing electropherograms confirming the targeted deletion.
